# Disrupted habenula function in major depression

**DOI:** 10.1038/mp.2016.81

**Published:** 2016-05-31

**Authors:** R P Lawson, C L Nord, B Seymour, D L Thomas, P Dayan, S Pilling, J P Roiser

**Affiliations:** 1 Institute of Cognitive Neuroscience, University College London, London, UK; 2 Wellcome Trust Centre for Neuroimaging, University College London, London, UK; 3 Computational and Biological Learning Lab, University of Cambridge, Cambridge, UK; 4 Centre for Information and Neural Networks, National Institute for Information and Communications Technology, Osaka, Japan; 5 Department of Brain Repair and Rehabilitation, University College London, London, UK; 6 Gatsby Computational Neuroscience Unit, University College London, London, UK; 7 Research Department of Clinical, Educational and Health Psychology, University College London, London, UK

## Abstract

The habenula is a small, evolutionarily conserved brain structure that plays a central role in aversive processing and is hypothesised to be hyperactive in depression, contributing to the generation of symptoms such as anhedonia. However, habenula responses during aversive processing have yet to be reported in individuals with major depressive disorder (MDD). Unmedicated and currently depressed MDD patients (*N=*25, aged 18–52 years) and healthy volunteers (*N*=25, aged 19–52 years) completed a passive (Pavlovian) conditioning task with appetitive (monetary gain) and aversive (monetary loss and electric shock) outcomes during high-resolution functional magnetic resonance imaging; data were analysed using computational modelling. Arterial spin labelling was used to index resting-state perfusion and high-resolution anatomical images were used to assess habenula volume. In healthy volunteers, habenula activation increased as conditioned stimuli (CSs) became more strongly associated with electric shocks. This pattern was significantly different in MDD subjects, for whom habenula activation decreased significantly with increasing association between CSs and electric shocks. Individual differences in habenula volume were negatively associated with symptoms of anhedonia across both groups. MDD subjects exhibited abnormal negative task-related (phasic) habenula responses during primary aversive conditioning. The direction of this effect is opposite to that predicted by contemporary theoretical accounts of depression based on findings in animal models. We speculate that the negative habenula responses we observed may result in the loss of the capacity to actively avoid negative cues in MDD, which could lead to excessive negative focus.

## Introduction

Major depressive disorder (MDD) is associated with problems exploiting affective information to guide goal-directed behaviour.^1^ Symptoms relating to motivational processing, such as anhedonia and fatigue, result in poorer treatment prognosis,^2, 3^ and standard treatments for depression are relatively ineffective in ameliorating them.^4^ The neurobiological mechanisms underlying motivational symptoms in depression remain poorly understood; however, contemporary theoretical accounts have suggested that they may be driven by hyperactivity in the habenula,^5^ a small brain structure adjacent to the medial dorsal (MD) thalamus that plays a central role in negatively motivated behaviour.

The habenula is extensively connected with the dorsal and medial raphe nuclei and ventral tegmental area (VTA), the sources of the brain's serotonin and dopamine neurons, respectively.^6, 7^ The afferent and efferent connections of the habenula (outlined comprehensively in a recent review^8^) suggest a route by which the habenula might affect monoaminergic transmission and thereby affective processing. Moreover, single-cell recording studies in non-human primates have demonstrated that the lateral habenula (LHb) responds to aversive stimuli,^9^ and that LHb stimulation profoundly inhibits VTA dopamine neuron firing.^10^ Thus, when habenula activity is high, dopamine activity is suppressed. Furthermore, optogenetic work in rodents provides convincing evidence that activating the habenula can promote various forms of behavioural avoidance.^11^ Therefore, LHb dysfunction might influence the processing of motivationally salient stimuli and thereby result in abnormal affective experience and behaviour.

Evidence supporting a role for the LHb in MDD comes from rodent models that use unavoidable aversive stimuli to induce learned helplessness.^12^ LHb metabolism is elevated in learned helplessness,^13^ and also in rats congenitally vulnerable to this procedure even before aversive stimulation.^14^ More recent studies have shown enhanced excitatory inputs to VTA-projecting habenula neurons in learned helplessness models,^15^ and that both elevated LHb spiking and depressive behaviours are reversed following antidepressant treatment.^16^ In addition, a complete stereotaxic pharmacological inhibition of the habenula has been reported to ameliorate depressive-like behaviour in a model of treatment-resistant depression.^17^ Furthermore, mice with dorsal medial habenula lesions show difficulty with performing motivation-based locomotor behaviours and show some depressive phenotypes related to hedonic state.^18^ These rodent studies suggest that habenula hyperactivity may contribute to depression, specifically its motivational components, and indeed some investigators have even tested whether deep brain stimulation to this structure can relieve symptoms in humans.^19, 20^ However, direct evidence for habenula hyperactivity in human depressed subjects is lacking.

Investigating the habenula noninvasively in humans poses a methodological challenge. Post-mortem^21^ and structural imaging data^22^ indicate that the human habenula is ∼15–30 mm^3^ in volume, less than the size of a typical voxel in functional magnetic resonance imaging (fMRI). In addition, standard pre-processing steps including normalisation and substantial spatial smoothing likely lead to localisation errors,^23^ making the signal from the habenula difficult to resolve from neighbouring structures. An early positron emission tomography study reported that in remitted MDD, Hamilton Depression Rating Scale (HAM-D) scores following tryptophan depletion correlated with resting-state blood flow in the vicinity of the habenula;^24^ consistent results were observed in a subsequent study using arterial spin labelling (ASL).^25^ Reduced habenula volume has also been reported in female MDD subjects,^22^ and a recent study reported that habenula glucose metabolism was decreased in MDD following ketamine treatment.^26^ However, no study to date has directly compared habenula function between currently depressed individuals and healthy volunteers (HVs) or explored the association between habenula structure or function and motivational processing.

In a recent experiment using computational modelling and high-resolution fMRI, we demonstrated that in HVs the habenula encodes the negative motivational value of conditioned stimuli (CSs), with greater activation elicited as the expectation of receiving a painful electric shock increases.^27^ Here we employed the same procedure to test our primary hypothesis that the habenula is phasically hyperactive (that is, in response to specific stimuli) in MDD. We additionally acquired high-resolution cerebral perfusion (ASL) images during the resting state to test our secondary hypothesis that the habenula is tonically hyperactive (that is, in the absence of specific external stimulation) in MDD. To confirm prior reports of reduced habenula volume in MDD,^22^ we quantified habenula volume on high-resolution anatomical images. Finally, we assessed whether habenula function and structure relate to the core motivational symptoms of depression using measures of anhedonia and fatigue.

## Materials and methods

### Participants

A total of 27 individuals meeting DSM-IV (Diagnostic and Statistical Manual of Mental Disorders, 4th Edition) criteria for MDD and 29 HVs group matched for age, gender and intelligence quotient (assessed by the Wechsler Test of Adult Reading) were recruited to participate in the study. Following exclusions (see Supplementary Methods for details), 25 subjects in each group were included in the analyses (males: MDD=15, HV=14).

Demographic and clinical data are displayed in Table 1 and full recruitment and exclusion criteria are included in the Supplementary Methods. All participants provided written informed consent and were compensated financially. The London Queen Square Research Ethics Committee approved the study (reference 10\H0716\2).

### Symptom measures

To assess motivational symptoms participants completed the Snaith–Hamilton Pleasure Scale (SHAPS)^28^ and the Fatigue Severity Scale (FSS):^29^ on both scales, higher scores indicate more severe symptoms. General depressive symptoms were assessed with the HAM-D^30^ and the Beck Depression Inventory (BDI).^31^

### Conditioning paradigm

**Figure 1:**
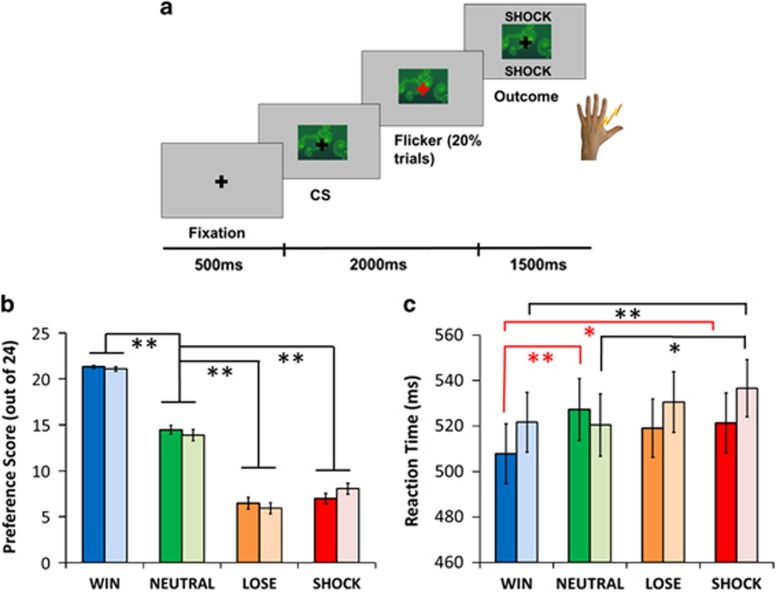
Conditioning task and results. (**a**) Exemplar trial (a detailed description is provided in the main text). (**b**) Explicit preference scores for win, loss, shock and neutral conditioned stimuli (CSs; maximum score of 24). (**c**) Reaction times to respond to fixation flickers overlaid on win, loss, shock and neutral CSs. Pale-coloured bars represent major depressive disorder (MDD) patients and bright-coloured bars represent healthy volunteers (HVs). Red and black solid lines show significant differences discussed in the text for the MDD and HV groups, respectively. Beyond the results discussed in the text, there was a significant main effect of cue type in the HVs (F(2.06, 49.66)=5.28, *P*=0.002) and a trend in the MDD group (F(3, 72)=2.42, *P*=0.073). Error bars show s.e.m. *P<0.05 and **P<0.001.

During fMRI, subjects performed a Pavlovian conditioning task during which they were passively exposed to seven CSs (abstract images) that were followed by different reinforcing outcomes (with high or low probability of reinforcement: win £1, lose £1 or painful electric shock, with the non-reinforced outcome being neutral; or a guaranteed neutral outcome) (Figure 1a and Supplementary Methods). During conditioning, subjects performed a fixation cross-flicker detection task to ensure attention (20% of trials, overlaid on CSs) that was independent of reinforcement. Three blocks were performed with different CSs in each block.

### Preference task

After each conditioning block, subjects' explicit knowledge of CS associations was assessed using a preference task, involving forced choices between pairs of CSs. See Supplementary Methods for further details.

### MRI acquisition and analysis

High-resolution (1.5 mm isotropic) fMRI data were acquired with a 3T Magnetom TIM Trio scanner (Siemens Healthcare, Erlangen, Germany) and analysed using Statistical Parametric Mapping (SPM8; www.fil.ion.ucl.ac.uk/spm).

For the analysis of habenula responses, we used a model-based fMRI approach^27, 32^ exploiting a reinforcement learning algorithm (with a predetermined learning rate of α=0.5)^33^ to calculate the trial-by-trial associative values of CSs that probabilistically predicted wins, losses and shocks. We then used these values in the fMRI analysis as parametric regressors whose onsets were time locked to the presentation of win, loss and shock CSs. Win, loss and shock outcomes were modelled in separate regressors. Flicker trials and any other trial on which subjects made a response were modelled separately. Cardiac and respiration parameters were also included as regressors to correct for physiological noise. We ran our model-based fMRI analyses across a range of learning rates (0.3–0.7) to ensure that our results were robust (Supplementary Results) as recommended for model-based fMRI analyses.^33^ See Supplementary Methods for further details relating to image acquisition, pre-processing and analysis.

Because our central hypotheses relate to the habenula, and given the small size of this structure, we manually defined regions of interest on high-resolution anatomical scans for the left and right habenula in each subject according to an established protocol^23^ (Supplementary Methods).

### ASL acquisition methods and processing

Cerebral blood flow (CBF) was measured using a pulsed ASL sequence.^34^ Raw CBF values were extracted from the habenula regions of interest of each subject and normalised for average whole volume perfusion. Further details are provided in the Supplementary Methods.

### Statistical analysis

Behavioural and habenula data were analysed in SPSS 20 (IBM, Chicago, IL, USA). All data were inspected before analysis to check for deviations from Gaussian distributions. Differences between conditions were analysed using repeated-measures analysis of variance with group as the between-subjects factor. Planned comparisons were conducted using independent samples *t*-tests (two tailed unless otherwise stated) between groups and paired samples *t*-tests within groups. Where assumptions of heterogeneity of covariance were violated, degrees of freedom were corrected using the Greenhouse–Geisser approach. Relationships between habenula structure and function and motivational symptoms were assessed using multiple linear regression.

### Power analysis

With 25 participants in the HV group we had 80% power to replicate the effect we observed previously for habenula responses to parametric shock CS values (*d* ~0.5)^22^ at α=0.05 (one tailed). With 25 depressed participants we had 80% power to detect a group difference of *d* ~0.8 at α=0.05 (two tailed).

## Results

### Behaviour

We confirmed conditioning by measuring relative CS preference. Consistent with our previous results, shock CSs were less preferred than win and neutral CSs (main effect of CS type: F(2, 96.16)=273.46, *P*<0.001). There was no group-by-stimulus interaction (F(2, 96.16)=0.85, *P*=0.471) suggesting that MDD and control participants learned the CS–outcome associations similarly (Figure 1b).

Reaction times to respond to the fixation cross flicker were longest on the negative (loss/shock) CS trials (main effect of CS type: F(3, 144)=3.68, *P*=0.014). The groups did not differ in terms of overall reaction times (main effect of group: F(1, 48)=0.22, *P*=0.641; Figure 1c), but there was a significant group × CS type interaction (F(3, 144)=3.14, *P*=0.027). Planned comparisons revealed that in both groups responses on win CS trials were faster than on shock CS trials (HVs: *t*(24)=3.30, *P*=0.003; MDD: *t*(24)=2.17, *P*=0.04). However, only the HVs responded faster on win relative to neutral CS trials (conditioned invigoration: *t*(24)=3.97, *P*=0.001), with a trend towards a difference between shock and neutral CS trials (conditioned suppression: *t*(24)=1.75*, P*=0.09). By contrast, MDD patients responded slower on shock relative to neutral CS trials *t*(24)=2.45, *P*=0.022), with no difference between win and neutral CS trials (*t*(24)=0.14, *P*=0.888).

### Phasic (stimulus-evoked) habenula function

**Figure 2:**
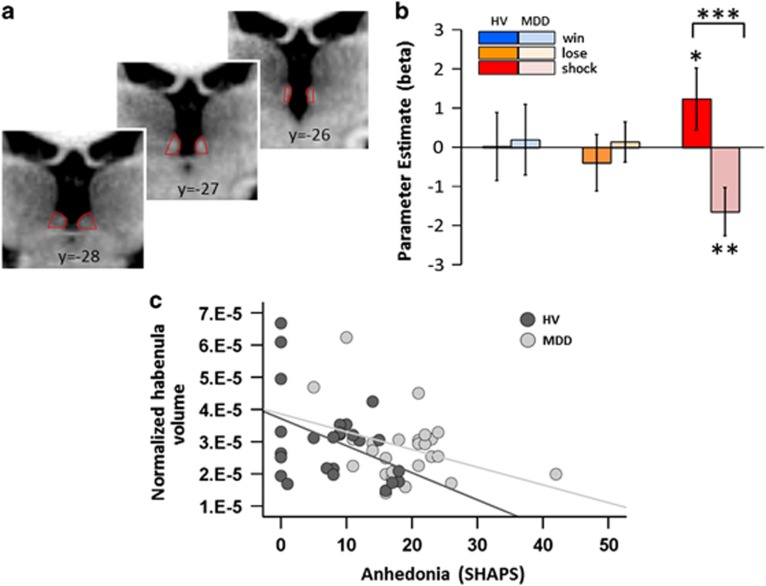
Habenula functional and anatomical data. (**a**) Location of the habenula on coronal slices of a representative subject. (**b**) Habenula response to the dynamically changing value of shock conditioned stimuli (CSs) is positive in healthy volunteers (HV) (bright red bar) and negative in major depressive disorder (MDD) participants (pale red bar). For the other CSs, pale-coloured bars represent MDD patients and bright-coloured bars represent HVs. These β-values represent the change in habenula response with increasing cue value. (**c**) The relationship between normalised average habenula volume and symptoms of anhedonia (Snaith–Hamilton Pleasure Scale (SHAPS)) in MDD participants (grey: *r*=−0.36, *P*=0.074) and HVs (black: *r*=−0.41, *P*=0.043). *P<0.07 (one tailed), **P<0.05 and ***P<0.001.

Next we analysed blood oxygen level-dependent (BOLD) signals in the habenula (Figure 2a), at the time of the CS, corresponding to computationally derived trial-by-trial fluctuation in CS values. The resulting β-values represent the strength of the regression coefficient relating changing CS value to habenula response.

A repeated-measures analysis of variance with hemisphere (left, right) and CS type (win, lose, shock) as within-subjects factors and group as a between-subjects factor revealed no main effect of hemisphere (F(1, 48)=2.05, *P*=0.159), no CS type-by-hemisphere interaction (F(2, 96)=0.31, *P*=0.735) and no group-by-CS type-by-hemisphere interaction (F(2, 96)=0.88, *P*=0.735). We identified no main effect of cue (F(2, 96)=0.12, *P*=0.883) and no main effect of group (F(1, 48)=0.97, *P*=0.329), but there was a significant CS type-by-group interaction (F(2, 96)=4.02, *P*=0.021).

Planned comparisons demonstrated that the habenula response to shock CS value in HVs was significantly greater than in the MDD group (*t*(48)=2.87, *P*=0.006, Figure 2b). As expected, the habenula response to parametrically varying shock CS value was positive in HVs, and showed a trend towards differing from zero (*t*(24)=1.55, *P*=0.066 (one tailed). Interestingly, two HVs scored in the range for mild–moderate depression on the BDI despite not fulfilling diagnostic criteria; and, when excluded, the habenula response to shock CS value achieved statistical significance (*t*(22)=1.96, *P*=0.03 (one tailed)). In other words, as observed in our previous study in an independent sample of HVs,^27^ the present data also show that as the CSs became more shock predicting, the response in the habenula increased. By contrast, habenula response to the value of shock CSs was significantly negative in the MDD patients (*t*(24)=2.67, *P*=0.01). Specifically, as the CSs became more shock predicting, the response in the habenula *decreased* in MDD patients.

There was no group difference in habenula responses to win or loss CS values (win: *t*(48)=0.13, *P*=0.891; loss: *t*(48)=0.60, *P*=0.551). Consistent with our previous study, habenula responses to win and loss CS values were not significantly different from zero in HVs (win: *t*(24)=0.03, *P*=0.977; loss: *t*(24)=0.54, *P*=0.592), and the same pattern was observed in MDD patients (win: *t*(24)=-0.22, *P*=0.829; loss *t*(24)=0.27, *P*=0.786). Exploratory voxel-wise contrasts of group differences in responses to parametric win, loss and shock CS values are presented in the Supplementary Table S1.

### MD thalamus responses

To exclude the possibility that signal from the MD thalamus, a comparatively large structure adjacent to the habenula, could be contributing to our effects we used the same bilateral MD thalamus regions of interest as employed in our previous investigation of the habenula in healthy volunteers.^27^ BOLD responses to the computationally derived values of win, loss and shock CSs were extracted from the left and right MD thalamus. There was no main effect of CS type (F(2, 96)=2.38, *P*=0.097), no main effect of hemisphere (F(1, 48)=0.97) and no main effect of group (F(1, 48)=1.30, *P*=0.25). Crucially the group-by-hemisphere (F<1), group-by-CS type (F(2, 96)=1.10, *P*=3.34), hemisphere-by-CS type (F<1) and three-way group-by-hemisphere-by-CS type interactions (F<1) were all nonsignificant.

### Tonic (resting-state) habenula function

**Figure 3:**
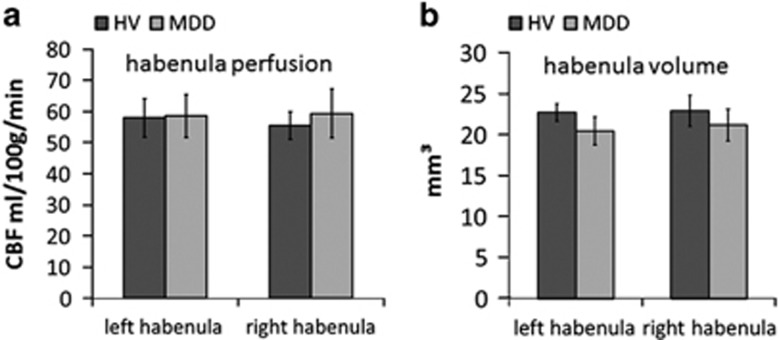
(**a**) Cerebral blood flow (CBF) values show no group difference in resting-state habenula blood flow. (**b**) Habenula volumes are not significantly different between the groups. Error bars represent s.e.m. HV, healthy volunteer; MDD, major depressive disorder.

Average habenula raw CBF was 58.97 ml per 100 g per min (s.d. 15.71) in the MDD subjects and 56.71 ml per 100 g per min (s.d. 19.10) in the HVs. Analysis of CBF (corrected for whole volume perfusion) revealed no main effect of hemisphere (F(1, 43)=0.20, *P*=0.647), no main effect of group (F(1, 43)=0.003, *P*=0.954) and no group-by-hemisphere interaction (F(1, 43)=0.834, *P*=0.366; Figure 3a). Exploratory voxel-wise contrasts of group differences in normalised CBF are presented in Supplementary Table S2.

### Habenula structure

Average habenula volume was 20.83 mm^3^ (s.d. 7.31) in the MDD patients and 22.31 mm^3^ (s.d. 9.29) in HVs. Analysis of habenula volume, corrected for whole brain grey matter, revealed no main effect of hemisphere (F(1, 50)=0.148, *P*=0.7), no main effect of group (F(1, 50)=0.091, *P*=0.764) and no hemisphere-by-group interaction (F(1, 50)=1.05, *P*=0.31; Figure 3b).

### Relationship between habenula structure/function and motivational symptoms

In order to test the hypothesis that habenula abnormalities drive motivational symptoms in MDD we constructed two multiple linear regression models, predicting anhedonia (SHAPS) and fatigue (FSS) respectively. These models used habenula BOLD responses to win, loss and shock CS values, normalised habenula CBF and normalised habenula volume as predictors, and both included group as a covariate. The model for SHAPS was significant (F(6, 38)=7.56, *P*<0.001, *r*^2^=0.544): group (patients scored higher: *t*(44)=4.96, *P*<0.001) and habenula volume (negative: *t*(44)=2.79, *P*=0.008) were significant predictors of anhedonia (higher anhedonia corresponding to smaller volume; Figure 2c), with no other predictors approaching significance (all *P*s>0.4). The group-by-habenula volume interaction was nonsignificant (*P>*0.5). The model for FSS was also significant (F(6, 38=12.34, *P*<0.001, *r*^2^=0.661): group was a significant predictor of fatigue (patients scored higher: *t*(44)=6.98, *P*<0.001) and there were trends for habenula CBF (positive: *t*(44)=1.83, *P*=0.074) and habenula BOLD response to win CS value (negative: *t*(44)=1.70, *P*=0.097), with no other predictors approaching significance (all *P*s>0.6). The interactions with group were nonsignificant (*P>*0.1).

Exploratory *post hoc* analyses investigating the relationship between habenula structure/function, general depressive symptoms and behaviour are presented in the Supplementary Results.

## Discussion

The results of this study suggest that MDD participants have abnormal phasic habenula responses elicited by cues that predict upcoming punishment. This difference cannot be accounted for by group differences in tonic habenula function or grey matter volume. In addition, smaller habenula volume was associated with greater anhedonic symptoms in both groups.

### Phasic habenula function

Our task-related measurements confirm that, in HVs, as CSs become more shock predicting, the habenula response increases.^27^ Although we note that this effect narrowly missed statistical significance in the full sample of HVs, our confidence in this finding is bolstered not only by the fact that it replicates our previous result in an independent sample,^27^ but also that when we excluded two HVs who scored in the mild–moderate range for depression on the BDI, the habenula response to shock CS value achieved statistical significance. Surprisingly, however, in MDD patients habenula responses *decreased* as CSs became more shock predicting (Figure 2b). The effect size for this finding is large (Cohen's *d* for the group difference in habenula response to shock CS value=0.82), highly anatomically specific (pertaining only the shock CS value cues in the habenula, and not the neighbouring MD thalamus) and robust to changes in learning rates (Supplementary Results).

This finding is contrary to hypotheses about phasic habenula function in MDD derived from animal studies exploring tonic habenula function that have been interpreted as indicating that the habenula is hyperactive in depression.^13, 14^ Recent studies in rodents have shown that excitatory synapses on to VTA-projecting LHb neurons are potentiated in learned helplessness,^15^ and one study reported a shift towards enhanced GABA/glutamate ratio in LHb synapses of rodents chronically treated with citalopram.^35^ Although these studies represent significant advances in understanding how animal LHb circuitry is altered by learned helplessness, to our knowledge there is currently no study directly examining aversive stimulus-evoked habenula firing in animal models of depression. We note that there is relevant work in primates,^36^ although not in the context of models of depression, and future work of this type assessing habenula responding during behaviour will be crucial in bridging the gap between human and animal research. However, it should be acknowledged that we did not identify abnormal habenula resting-state perfusion in MDD patients in the present study, although the ASL measurements were always acquired after the conditioning task, and it is therefore possible that perfusion may have been affected by the prior aversive stimulation.

The surprising result that the habenula response to shock-predicting CSs in MDD is in the opposite direction to that identified in HVs across two studies requires substantial further study. One possibility is that this opposite response leads to a loss of capacity for active avoidance, specifically for primary punishments. This suggestion is consistent with recent optogenetic studies that demonstrate that activation of the LHb promotes active behavioural avoidance of stimuli associated with negative consequences.^11, 37^ We note here that our behavioural data speak only to passive avoidance (conditioned suppression) that does seem to be spared in MDD (Figure 1b).

Consistent with our previous study,^27^ habenula responses to win and loss CSs in HVs did not differ from zero in either group (Figure 2b). Similarly, animal data suggest that the habenula is a ‘reward-negative' brain region, predominantly concerned with primary aversive outcomes and the cues that predict them.^9^ In humans, negative feedback, as well as self-detected errors, have been reported to increase BOLD response in the vicinity of the habenula (although not yet measured at high resolution).^38^ In further agreement, the only other high-resolution fMRI study of the human habenula also failed to detect responses to reward predicting CSs.^39^ Indeed, it is possible that in the present study electric shocks (primary punishments) framed the task such that all nonshock outcomes (that is, win and loss; secondary reinforcers) were less motivationally salient, attenuating neural responses to their associated CSs. In our previous investigation, this interpretation was supported by pupil data, an implicit measure of conditioning, which showed greater dilation for shock-predictive CSs relative to all other CSs, despite explicit preference scores showing no difference between the primary and secondary punishments;^27^ however, in the present study we were unable to collect pupil data because of time constraints in the scanner.

Should we interpret increased habenula BOLD activity as a mark of increased activity of the output cells of the habenula? The positive BOLD response to increasingly aversive CSs seen here in HVs and also previously^27^ aligns with the spiking activity of habenula neurons in non-human primates in response to negative outcomes and cues that predict those outcomes.^9^ This would be consistent with the interpretation that opposite habenula response, seen here in MDD, does indeed reflect reduced habenula firing in response to the strength of shock-predicting cues. However, the BOLD signal may reflect synaptic input into a region,^40^ whereas animal research investigating habenula function in learned helplessness has typically explored efferent synaptic connections between the habenula and the VTA.^15^ In addition, BOLD activity may reflect local inhibition,^41^ and the relationship between BOLD response and spiking activity can vary markedly across the brain.^42^ Furthermore, even with 1.5 mm isotropic voxels, we do not have sufficient resolution to disambiguate the medial and lateral portions of the habenula, and it is possible that signal from the medial habenula contributes to the disrupted response in MDD patients that we identified here. In addition, rodent studies of gene expression in the habenula reveal a striking variety of neuron types^43^ that may be differentially affected in MDD and could affect the BOLD signal in different ways. Indeed, even within the lateral portion of the habenula there appears to be heterogeneity, as a very small number of the cells there seem to drive the behaviours observed in rodent models of depression.^16^ Nonetheless, high-resolution fMRI offers the only noninvasive method to investigate the function of this structure in humans, and provides a vital link from animal models to clinical symptoms in humans. These results highlight the need for greater synergy across human and animal research and the integration of findings at both micro and macro scales.

### Tonic habenula function

A few previous studies in humans have suggested that the habenula may be tonically hyperactive in MDD.^24, 25^ By contrast, we detected no group differences in normalised CBF as measured by ASL (Figure 3a). We note that in both these previous reports elevated habenula perfusion was identified only under conditions of tryptophan depletion, complicating their interpretation. In addition, neither of them was conducted at high resolution, raising the possibility that perfusion measurements may have been contaminated by nearby structures. A recent positron emission tomography imaging study (with 6.5 mm resolution) has shown decreased glucose metabolism in the vicinity of the habenula following treatment with ketamine,^26^ and this is of interest in light of two studies suggesting that ketamine specifically ameliorates anhedonia, over and above the overall improvement in depressive symptoms.^44, 45^ However, energy consumption in the brain reflects both inhibitory and excitatory demands that cannot easily be differentiated by positron emission tomography, and hence this finding does not conclusively speak to the status of tonic habenula function in MDD. Finally, two clinical case studies have reported remission of symptoms in treatment-resistant depression following deep brain stimulation of the habenula.^19, 20^ In the latter study, *increased* metabolism (measured by PET with 3.5 mm resolution) was apparent following deep brain stimulation in the vicinity of the habenula after 6 months of stimulation. Therefore, on balance of all available evidence to date, the hypothesis that increased tonic habenula activity drives symptoms in MDD is not, at present, well supported by either our current results or prior data in humans.

### Habenula structure

A previous study examining differences in habenula volume between MDD, bipolar depression (BD) and HVs reported lower habenula volume in BD, and exploratory analyses identified reduced habenula volume only in female MDD patients compared with female HVs.^22^ Although a large proportion of our sample was female, we did not find any group differences in habenula volume (Figure 3b); that said, average habenula volume was numerically lower in the MDD group than the HV group (mean: 1.99  mm^3^, s.e.: 2.36 mm^3^ difference). Interestingly, this numerical difference was greater in females (mean: 2.41 mm^3^, s.e.: 2.95 mm^3^ difference). Our study was not designed to assess this question, and this null result should be interpreted with caution. Indeed, in order to detect a difference in normalised habenula volume between female MDD and healthy volunteers of the magnitude reported by Savitz *et al.*^22^ (Cohen's *d* ~0.53), we would have required 57 female participants in each group to achieve 80% power.

Lower habenula volume was associated with symptoms of anhedonia in both groups in our sample (Figure 2c). However, we did not specifically aim to recruit an anhedonic MDD sample in this study and, in light of the inverse correlation between anhedonia and habenula volume, this suggests a possible reason for the lack of a significant group difference in habenula volume. It would be of interest in future studies to recruit groups of patients with specific symptom profiles (for example, with pronounced melancholic symptoms) to assess whether habenula abnormalities occur specifically in patients suffering from anhedonia. We note, however, that habenula volume was not a significant predictor of fatigue and, interestingly, that fatigue and anhedonia were not correlated in the MDD group (*r*=0.058, *P*=0.79). This suggests that anhedonia (loss of enjoyment or pleasure) uniquely predicts habenula volume over and above general tiredness, although both could be considered motivational symptoms.

At present, the microstructure and cellular events that give rise to grey matter volume as measured by structural MRI remain poorly understood,^46^ and future basic research in this area will be necessary before mechanistic interpretations can be applied to the correlations discussed above. Interestingly, a recent whole brain voxel-based morphometry study identified increases in habenula grey matter volume following treatment with electroconvulsive therapy in patients with treatment-resistant depression,^47^ although we mention this result with the caveat that an 8 mm smoothing kernel was applied to these data. Although this study reported no correlation between grey matter volume and clinical scores, future studies should examine the specific relationship between grey matter volume and motivational symptoms during treatment.

### Summary

We believe we conducted the first comprehensive investigation of habenula structure and function in MDD using a computational psychiatry approach,^48^ in combination with high-resolution neuroimaging. These data support the notion that habenula function is disrupted in MDD, but not the simple ‘hyperactivity' hypothesis.

## Figures and Tables

**Table 1:** Characteristics of the participants

*Measure*	*HV (*n=*25)*^a^	*MDD (*n=*25)*^b^	*Significance*
Age, mean (s.d.), years	27.44 (8.75)	27.76 (9.01)	0.90
Sex, *n* males	14/25	15/25	
HAM-D, mean (s.d.)	1.64 (1.52)	19.08 (3.75)	<0.001
BDI, mean (s.d.)	3.76 (4.13)	25.36 (8.94)	<0.001
FSS, mean (s.d.)	26.60 (8.76)	45.92 (9.46)	<0.001
SHAPS, mean (s.d.)	7.80 (6.35)	18.96 (7.02)	<0.001
Wechsler Test of Adult	110.92 (5.15)	110.12 (7.54)	0.66
Reading predicted FSIQ, mean (s.d.)			
Average first-deg rel with MDD, mean (s.d.)	NA	1.86 (1.30)	
Average first-deg rel treated, mean (s.d.)	NA	1.47 (1.25)	
Age first episode, mean (sd)	NA	18.16 (5.64)	
Total no. of episodes, mean (s.d.)	NA	2.88 (1.01)	
Past antidepressant use, *n*	NA	14/25	
Years since last medicated, mean (s.d.)	NA	6.6 (6.8)	
Hospitalised for MDD, *n*	NA	6/25	
Attempted suicide, *n*	NA	10/25	
Average no. of suicide attempts, mean (s.d.)	NA	1.08 (1.52)	
Shock level, mA (s.d.)	0.45 (0.19)	0.43 (0.53)	0.86
% Missing resp (s.d.)–conditioning task	3.14 (1.15)	4.17 (1.10)	0.52
			
*Movement parameters*			
Sum absolute translations, mm	12.80 (8.16)	10.03 (7.74)	0.30
Sum absolute rotation, deg	13.31 (11.00)	10.27 (11.22)	0.44

Abbreviations: BDI, Beck Depression Inventory; deg, degree; FSIQ, Full Scale Intelligence Quotient; FSS, Fatigue Sensitivity Scale; HAM-D, Hamilton Rating Scale for Depression; HV, healthy volunteer; MDD, major depressive disorder; NA, not available; rel, relative; resp, response; SHAPS, Snaith–Hamilton Pleasure Scale.

a A total of 23 in arterial spin labelling (ASL) analysis.

b A total of 22 in ASL analysis.
