# Supplementary material for: Disrupted habenula function in major depression
Source: Mol Psychiatry. 2016 May 31;22(2):202–8. doi: 10.1038/mp.2016.81 (PMC5285459; doi:10.1038/mp.2016.81)
Supplement: Supplementary Information [file mp201681x1.doc]

Supplementary Materials

1. **Supplemental methods**
2. **Supplemental Results**
3. **Supplemental Figure S1**
4. **Supplemental Figure S2**
5. **Table S1.** Exploratory whole-brain fMRI results
6. **Table S2**. Exploratory whole-brain ASL results
7. Supplemental Methods

**Participants**

Twenty-seven individuals meeting DSM-IV criteria for MDD and twenty-nine healthy volunteers were recruited to take part in the study. Data from one healthy volunteer were lost due to equipment failure and data from three further healthy volunteers were excluded due to diagnosis of neurological/psychiatric disorders. Two MDD patients were excluded due to movement-related corruption of images. This left data from 25 MDD patients and 25 controls for analysis. Demographic information is provided in Table 1.

MDD patients were recruited from Camden and Islington NHS Foundation Trust and via advertisement. Current and past psychiatric diagnosis was assessed using the Mini International Neuropsychiatric Interview (MINI)^1^. Inclusion criteria were a current major depressive episode and age 18-55 years. Exclusion criteria were: past/present neurological disorders; past/present manic or hypomanic episodes; past/present alcohol/substance dependence/abuse (save for a remote (>6 month) history of abuse); past/present psychotic disorder; use of psychotropic medication within 4 weeks of participation (8 for fluoxetine); MRI contraindications. MDD participants were not excluded on the basis of anxiety disorders. HVs were recruited via advertisement and also assessed with the MINI. Exclusion criteria were any past/present psychiatric or neurological disorders and MRI contraindications.

**Conditioning task**

We used a Pavlovian conditioning paradigm in which visual CSs (fractal images) were probabilistically paired with win, loss, shock or neutral outcomes ^2^. There were seven CSs, associated with the following outcomes: 75% chance of £1 win; 25% chance of £1 win; 75% chance of £1 loss; 25% chance of £1 loss; 75% chance of shock; 25% chance of shock; 100% chance of no outcome (neutral). On trials where the reinforcing outcome (win, lose or shock) was not presented, and on neutral trials, the word “nothing” was presented on screen. The task is presented in Figure 1a. On each trial subjects initially saw a fixation cross which remained on-screen for the entire trial; the CS appeared after 500ms, remaining on-screen until the end of the trial; and the outcome was presented 2000ms following the CS onset. To ensure attention, on 20% of trials the fixation cross in the centre of the screen flickered from black to red for 300 ms during CS presentation (but before outcome), and participants were instructed to respond via a button press whenever this occurred. They were explicitly instructed that their responses made no difference to the outcomes they received. These trials were excluded from functional magnetic resonance imaging (fMRI) analysis. In total 420 trials were presented over three blocks, each lasting 9.3 minutes. CSs were luminance matched and assigned to conditions randomly across subjects and across blocks. Since new stimuli were associated with each outcome in each block, new learning occurred within each block.

**Preference task**

After each conditioning block, subjects’ explicit knowledge of CS values was assessed using a preference task ^2^ involving forced choices between pairs of CSs. Each CS was paired four times with every other CS, and subjects indicated which one they preferred. The position of each CS (on the left or right side of the screen) was randomized. The total number of preference choices for each CS was summed to calculate an average preference score (out of 24).

**Pain calibration**

Electric shocks were delivered to the left hand (fascia over adductor pollicis muscle) via a silver chloride electrode, using a single 1000 Hz electrical pulse. Subjects underwent a thresholding procedure to control for heterogeneity in skin resistance and pain tolerance ^3^. Shocks were administered sequentially with step increases in amplitude and subjects provided visual analog ratings of each shock on a scale from “0 – not painful” to “10 – terrible pain/pain that would cause me to move in the scanner”. Shock intensity data are provided in Table 1.

**MRI acquisition & pre-processing**

Acquisition: MRI data were acquired with a 3T Magnetom TIM Trio scanner (Siemens Healthcare, Erlangen, Germany) fitted with a 32-channel radio-frequency receive head coil and body transmit coil. High-resolution T2*-weighted 2D echo-planar images (EPIs) were obtained using a custom-written sequence with the following parameters^4^: matrix size: 128x128; field-of-view (FOV): 192x192 mm; in-plane resolution: 1.5x1.5 mm; interleaved slice order acquisition; slice thickness: 1.5 mm with no gap between slices; excitation flip angle: 90°; echo time (TE): 36.2 ms; slice repetition time (TR): 84.2 ms; volume TR 3.2s. Thirty-eight slices were acquired with the FOV centered manually in-line with the habenula in each subject. After reconstruction three slices were discarded on either side of the encoding slab to avoid edge artifacts due to motion, leaving a total of 32 slices in each volume. Five dummy volumes were acquired prior to the image volumes to allow for T1 equilibration. Field-maps were also acquired. Cardiac pulse signal and respiration were measured during EPI runs using a pulse oximeter and a pneumatic belt respectively. These were used to correct for pulse- and respiration-related artefacts during analysis (see below) ^5^. High-resolution T1-weighted anatomical images were acquired using an optimized 3D MDEFT imaging sequence with correction for B1 in-homogeneities at 3T^6^. Image resolution was 770 μm isotropic (matrix size: 304 × 288 × 224; TR: 7.92 ms; TE: 2.48 ms; excitation flip angle: 16°).

Pre-processing: Statistical parametric mapping (SPM8; Wellcome Trust Centre for Neuroimaging, www.fil.ion.ucl.ac.uk/spm) was used to analyze all MRI data. For the ROI analysis of the habenula, each subject’s data were slice-time corrected, realigned to the first image, unwarped using a field-map of the static magnetic field (B0)^7^ and co-registered to their individual anatomical scan, on which the habenula ROIs were placed according to a previously described procedure ^2,8^. Images were smoothed using a Gaussian kernel with full width at half maximum (FWHM) of 2 mm to increase signal-to-noise without smoothing signal beyond the limits of the habenula ROI ^8^. Inter-scan movement was assessed in each participant and each run separately. Any scan in which participants moved more than one-half of a voxel in translation or rotation was removed from subsequent analysis and replaced with the average of surrounding scans, following visual inspection for artefacts. Runs requiring interpolation of >10% data were excluded from further analysis. There were no group differences in summed absolute translations or rotations (**Table 1**).

First-level analysis: We used a reinforcement learning model to generate inferred values for the win, loss and shock CSs on every trial^9^. Specifically, we used a temporal difference model in which the value (v) of a particular CS (referred to as a state (s)) is updated according to the following learning rule: v(s + 1) ←v(s) + αδ, where δ is the prediction error, defined as: δ = r – v(s), and r is the outcome received. In the absence of behavioural responses on every trail we are unable to estimate a learning rate from the data so we chose a learning rate of α=0.5, which is supported by a number of studies, examining both Pavlovian and instrumental learning^10,11^, and which we used in our prior study examining aversive learning in the habenula healthy volunteers ^2^. Fixed model parameters, such as learning rate, across all subjects and all groups have been justified in previous computational fMRI studies exploring group differences because neuroimaging analyses test the null hypothesis of no difference between groups ^12,13^. Additionally, we ran our model-based fMRI analyses across a range of learning rates (0.3-0.7) to ensure that our results were robust to learning rate differences as recommended for model-based fMRI analyses ^14^.

At the subject-level, fMRI data were analyzed in an event-related manner, using the general linear model, with the onsets of each win, loss and shock CSs (high and low probability stimuli combined in a single regressor) convolved with the SPM synthetic hemodynamic response function in separate regressors. We used the model-based fMRI approach, in which the computationally-derived CS values (see above paragraph) parametrically modulated the CS onset regressors on a trial-by-trial basis. We also included in the model regressors for the onsets of win, loss, shock and neutral outcomes, as well as realignment parameters to correct for subject movement, and cardiac and respiration parameters to correct for physiological noise. Note that our main inferences relate to the parametric regressors corresponding to the values of win, loss and shock CSs, which are orthogonal to the regressors they modulate.

Second-level analysis: Group-level contrasts used the standard summary-statistics approach to random-effects analysis in SPM. Contrast estimates representing the win, loss and shock CS values (i.e. the parametric modulator regressors from the subject-level) were extracted from each individual’s habenula ROI. Summary statistics conducted on these contrast estimates indicate the statistical reliability of the regression coefficient relating continuously varying CS value to habenula response and, as such, do not necessitate a baseline comparison. For the exploratory whole-brain analysis, the respective contrast images for each subject were normalized to the standard space Montreal Neurological Institute template using the Dartel toolbox for SPM^15^, smoothed with an 8-mm FWHM kernel, and included in between-groups two-sample *t*-tests thresholded at an exploratory threshold of *P* < 0.01 (*k* ≥ 20). Note that where we describe a CS as punishment or reward ‘predicting’ this is in reference to the expected value of the outcome of which the cue is predictive (e.g. our computationally derived regressors in the fMRI analysis).

**Arterial Spin Labelling acquisition methods & processing**

Cerebral blood flow (CBF) was measured using a pulsed arterial spin labelling (PASL) approach ^16^. A FAIR Q2TIPS labelling scheme was used in conjunction with a segmented 3D GRASE acquisition module ^17^. A 60 mm thick axial imaging slab was used, centred on the habenula. Acquisition parameters were: TI1=800 ms; TI2=1800 ms; background suppression (2 pulses); 8 shot interleaved acquisition with segmentation along the phase encode direction; 30 partitions; 5/8 partial Fourier along partition direction; 2x2x2 mm^3^ isotropic resolution; TE=13.72ms; TR=3s; 12 averages; total acquisition time = 9min 36s. A separate long TR acquisition was performed to obtain an M0 image for CBF quantification.

CBF quantification was performed using custom-written Matlab program. The difference between the ASL labelled and control images was normalised using the M0 image and CBF maps were calculated using the general kinetic model^18^, modified to account for the Q2TIPS labelling scheme ^19^. Due to time constraints and excessive specific absorption rate (SAR) levels in some participants, leading to safety concerns, ASL data were only available for 23/25 MDD patients and 22/25 controls.

Second-level analysis: CBF maps were co-registered to each individual’s structural image in native space and values were extracted from each individual’s habenula ROI and corrected for whole-brain perfusion. For the exploratory whole-brain analysis, the respective CBF maps for each subject were normalized to the standard space Montreal Neurological Institute template using the Dartel toolbox for SPM^15^ and included in between-groups two-sample *t* tests thresholded at an exploratory threshold of *P* < 0.01 (*k* ≥ 20).

**Volumetric Analysis**

Each subject's anatomical image was manually reoriented such that the coordinates [x = 0, y = 0, z = 0] occupied the midpoint of the anterior commissure (AC) with deviations from the origin being defined relative to the AC. Further, the brain was oriented along the AC–PC line passing through the centres of each commissure, after ^20^. The habenula was defined for each individual using anatomical landmarks according to our previously published protocol, which excludes the white matter structures adjoining the habenula and neighbouring grey matter structures such as the MD thalamus. Total habenula volume was taken as the summed volume of the voxels in each ROI using custom written scripts for SPM. Habenula volume measurements used in all subsequent analysis were normalised for whole brain grey matter as in previous volumetric studies of this structure ^21,22^. The results of regression analyses using these normalized measurements were unaffected when age was included as a covariate.

1. Supplemental Results

**Habenula ROI results at different learning rates.**

To exclude the possibility that our habenula fMRI result depends on the fixed learning rate (alpha=0.5), we ran our model-based analysis at a range of learning rates (alpha=0.3 and alpha=0.7; Supplemental Figure S1). Average habenula response at both alphas revealed a significant CS-by-group interaction (alpha=0.3, F(2,96)=3.64, P=0.03; alpha=0.7, F(2,96)=4.00, P=0.021). Crucially, the habenula response to shock CS value in MDD was significantly negative at both alphas (alpha=0.3, *t*(24)=2.35, *P*=0.027; alpha=0.7, *t*(24)=2.76, *P*=0.011) and significantly different to HVs at both alphas (alpha=0.3, *t*(48)=1.85, *P*=0.012; alpha=0.7, *t*(48)=2.97, *P*=0.005). The HV response to shock CS value was also positive at both alphas (alpha=0.3, *t*(24)=1.52, *P*=0.069 (1-tailed); alpha=0.7, *t*(24)=1.58, *P*=0.064 (1-tailed)). The habenula response to win and lose CS value was not significantly different from zero or significantly different between the groups at either alpha (all Ps>0.35). These results demonstrate that our group difference in habenula response to shock CS value is not substantially affected by the fixed learning rate.

**Habenula function and behaviour**

To explore the relationship between habenula function and behaviour we ran an exploratory multiple linear regression model predicting habenula response to shock CS value (our central fMRI result) with group and also four behavioural predictors (conditioned suppression, conditioned invigoration, preference for neutral cues, relative to shock cues, and preference for win cues, relative to neutral cues). This model was significant F(5,49)=2.53, P=0.042) and group was the only significant predictor (t(49)=2.25, P=0.018). Additionally there were no interactions with group. This demonstrates that there is no relationship between habenula response to shock CS value and RTs/preference scores. We note, however, that our primary hypotheses concerned the relationship between the habenula and motivational symptoms as we have reported in the manuscript.

**Habenula structure and depressive symptoms**To explore the relationship between habenula structure and depressive symptoms we tried a similar regression strategy to predict HAM-D and BDI scores from normalised habenula volume, but these analyses were difficult to interpret because the homogeneity of covariance assumption was violated in each case. Instead, we examined the correlation coefficients in each group separately which revealed that HAM-D positively predicted habenula volume in the MDD group (r²=0.734, p<0.001), but BDI did not (r²=0.338, p>0.09). In the HVs neither measure correlated significantly with habenula volume (*p*s>0.09). The direction of this relationship (high depressive symptoms predicts large habenula volume) is opposite to the relationship between habenula volume and SHAPS (high anhedonia predicts small habenula volume). While it is possible that depressive and anhedonic symptoms are associated with habenula volume in different ways, we note that this effect is driven by the four participants who scored highest on the HAM-D (Supplemental Figure S2), and that we had no *a priori* hypothesis about the relationship between the habenula and general depressive symptoms. Therefore we are reluctant to draw any firm conclusions on the basis of this exploratory finding.

1. Supplemental Figure S1


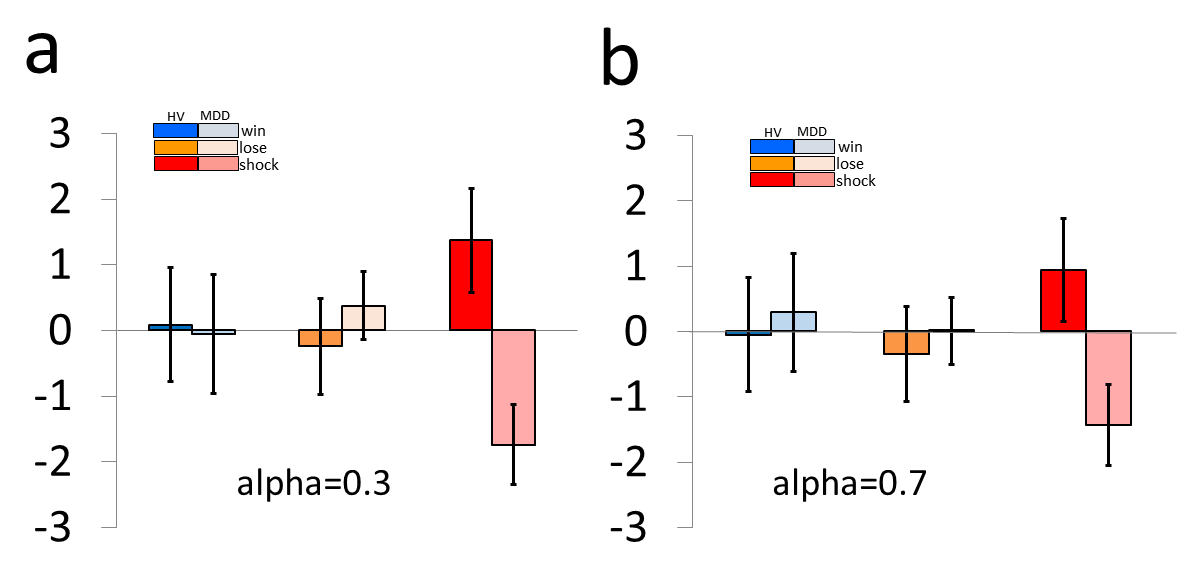


**Figure S1**: (a) Habenula ROI results at different learning rates. Similar results were obtained when using learning rates of (A) alpha = 0.3 and (B) alpha = 0.7 (original alpha = 0.5). Error bars represent SEM.

1. Supplemental Figure S2


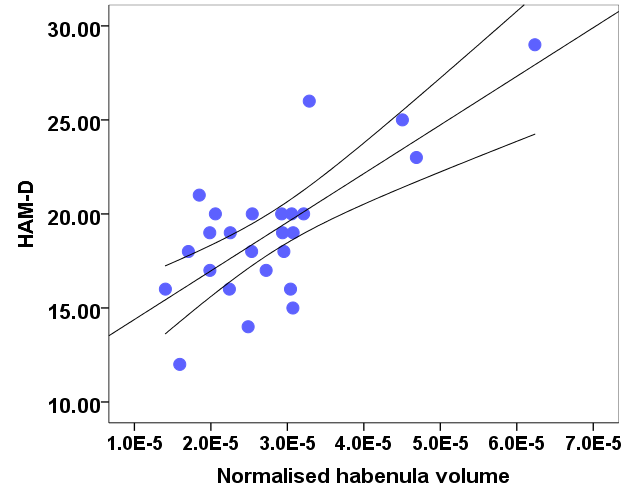


**Figure S2**: (a) The relationship between habenula volume and general depressive symptoms (Hamilton Depression Rating Scale: HAM-D). Note that this effect is driven by a small number of subjects who score highest on the HAM-D and as such this result should be interpreted with caution.

**References**

1 Lecrubier Y, Sheehan DV, Weiller E, Amorim P, Bonora I, Sheehan KH *et al.* The Mini International Neuropsychiatric Interview (MINI). A short diagnostic structured interview: reliability and validity according to the CIDI. *Eur Psychiatry* 1997; **12**: 224–231.

2 Lawson RP, Seymour B, Loh E, Lutti A, Dolan RJ, Dayan P *et al.* The habenula encodes negative motivational value associated with primary punishment in humans. *Proc Natl Acad Sci* 2014; **111**: 11858–11863.

3 Vlaev I, Seymour B, Dolan RJ, Chater N. The Price of Pain and the Value of Suffering. *Psychol Sci Wiley-Blackwell* 2009; **20**: 309–317.

4 Lutti A, Thomas DL, Hutton C, Weiskopf N. High‐resolution functional MRI at 3 T: 3D/2D echo‐planar imaging with optimized physiological noise correction. *Magn Reson Med* 2013; **69**: 1657–1664.

5 Hutton C, Josephs O, Stadler J, Featherstone E, Reid A, Speck O *et al.* The impact of physiological noise correction on fMRI at 7 T. *NeuroImage* 2011; **57**: 101–112.

6 Deichmann R. Fast structural brain imaging using an MDEFT sequence with a FLASH-EPI hybrid readout. *NeuroImage* 2006; **33**: 1066–1071.

7 Hutton C, Bork A, Josephs O, Deichmann R, Ashburner J, Turner R. Image Distortion Correction in fMRI: A Quantitative Evaluation. *NeuroImage* 2002; **16**: 217–240.

8 Lawson RP, Drevets WC, Roiser JP. Defining the habenula in human neuroimaging studies. *NeuroImage* 2013; **64**: 722–727.

9 O’Doherty JP, Dayan P, Friston K, Critchley H, Dolan RJ. Temporal Difference Models and Reward-Related Learning in the Human Brain. *Neuron* 2003; **38**: 329–337.

10 Seymour B, O’Doherty JP, Koltzenburg M, Wiech K, Frackowiak R, Friston K *et al.* Opponent appetitive-aversive neural processes underlie predictive learning of pain relief. *Nat Neurosci* 2005; **8**: 1234–1240.

11 Seymour B, Daw N, Dayan P, Singer T, Dolan R. Differential Encoding of Losses and Gains in the Human Striatum. *J Neurosci* 2007; **27**: 4826–4831.

12 Kumar P, Waiter G, Ahearn T, Milders M, Reid I, Steele JD. Abnormal temporal difference reward-learning signals in major depression. *Brain* 2008; **131**: 2084–2093.

13 Pessiglione M, Seymour B, Flandin G, Dolan RJ, Frith CD. Dopamine-dependent prediction errors underpin reward-seeking behaviour in humans. *Nature* 2006; **442**: 1042–1045.

14 Wilson R, Niv Y. Is Model Fitting Necessary for Model-Based fMRI? *PLoS Comput Biol* 2005; **11**. doi:doi: 10.1371/journal.pcbi.1004237.

15 Ashburner J. A fast diffeomorphic image registration algorithm. *NeuroImage* 2007; **38**: 95–113.

16 Wong EC. An introduction to ASL labeling techniques. *J Magn Reson Imaging* 2014.

17 Feinberg D, Ramanna S. Evaluation of new ASL 3D GRASE sequences using parallel imaging, segmented and interleaved K-space at 3T with 12-and 32-channel coils. In: *Proc. Intl. Soc. Mag. Reson. Med*. 2009, p 623.

18 Buxton RB, Frank LR, Wong EC, Siewert B, Warach S, Edelman RR. A general kinetic model for quantitative perfusion imaging with arterial spin labeling. *Magn Reson Med* 1998; **40**: 383–396.

19 Wong EC, Buxton RB, Frank LR. Quantitative imaging of perfusion using a single subtraction (QUIPSS and QUIPSS II). *Magn Reson Med* 1998; **39**: 702–708.

20 Mai J., Paxinos G, Voss T. *Atlas of the Human Brain*. 3rd ed. Academic Press: Elsevier, 2008.

21 Savitz JB, Nugent AC, Bogers W, Roiser JP, Bain EE, Neumeister A *et al.* Habenula volume in bipolar disorder and major depressive disorder: a high-resolution magnetic resonance imaging study. *BIOL PSYCHIATRY* 2011; **69**: 336–343.

22 Savitz J, Bonne O, Nugent A, Vythilingam M, Bogers W, Charney D *et al.* Habenula volume in post-traumatic stress disorder measured with high-resolution MRI. *Biol Mood Anxiety Disord* 2011; **1**: 7.

23 Kriegeskorte N, Bandettini P. Analyzing for information, not activation, to exploit high-resolution fMRI. *Neuroimage* 2007; **38**: 649–662.

1. Table S1

**Table S1**: Whole-brain analysis. Activations derived from group comparisons of contrasts corresponding to win CS, loss CS, and shock CS values (exploratory cluster-forming threshold *p*<0.01, k≥20). HV = healthy volunteer; MDD = major depressive disorder; k=cluster size; MNI=Montreal Neurological Institute; Z=Z statistic. BA=Brodmann area. Note that due to the multiple comparisons problem posed by high-resolution data ^23^ the information in this table is provided for information only.

1. Table S2

**Table S2**: Whole-brain ASL. Group comparisons pertaining to CBF (exploratory cluster-forming threshold *p*<0.01, k≥20). Abbreviations as in Table 1.
